# Task-guided accelerated cTBS simultaneously treats depression and social dysfunction in patients with major depressive disorder: a randomized clinical trial

**DOI:** 10.1038/s41386-026-02365-7

**Published:** 2026-02-20

**Authors:** Jing Jin, Yun Wang, Pengfei Wang, Qin Qin, Zhimin Wang, Yuening Jin, Xiongying Chen, Yuan Feng, Paul B. Fitzgerald, Peter Zeidman, Yuan Zhou, Gang Wang

**Affiliations:** 1 National Clinical Research Center for Mental Disorders & National Center for Mental Disorders, Beijing Anding Hospital, Capital Medical University, Beijing, China; 2 Advanced Innovation Center for Human Brain Protection, Capital Medical University, Beijing, China; 3 State Key Laboratory of Cognitive Science and Mental Health, Institute of Psychology, Chinese Academy of Sciences, Beijing, China; 4 Department of Psychology, University of Chinese Academy of Sciences, Beijing, China; 5 School of Medicine and Psychology, College of Science and Medicine, The Australian National University, Canberra, Australia; 6 Wellcome Centre for Human Neuroimaging, University College London, London, UK

**Keywords:** Depression, Translational research

## Abstract

Current antidepressant therapies predominately target symptom remission in major depressive disorder (MDD) but often overlook social dysfunction, which is a critical determinant of functional recovery. Individualized, accelerated continuous theta burst stimulation (cTBS) targeting the right dorsolateral prefrontal cortex (R.DLPFC) may offer a potential therapeutic approach for concurrently improving clinical and psychosocial outcomes in MDD. In this randomized, double-blind, sham-controlled trial, 70 patients with MDD were randomly assigned to receive either active (*n* = 37) or sham (*n* = 33) accelerated cTBS for 2 weeks. The treatments targeted individualized R.DLPFC sites, which were identified by task-evoked brain activation during a social interactive task (Ultimatum Game, UG). Depressive and anxiety symptoms, general social functioning, UG-derived behavioral and computational modeling indices (e.g., learning rates), and effective connectivity in social processing networks were assessed pre- and post-treatment. Active accelerated cTBS significantly outperformed sham stimulation in reducing depressive and anxiety symptoms and improving general social functioning (all *ps* < 0.001). The active group also demonstrated enhanced cooperation behavior (*p* = 0.002) and increased learning rates (89% HDI: [0.01, 0.21]). And the active group exhibited increased effective connectivity from the right insula to the R.DLPFC and stable effective connectivity from the anterior cingulate cortex (ACC) to the left insula after treatment, which was different from the sham group (*Pp* > 0.95). No severe adverse events occurred. Individualized, accelerated cTBS targeting task-evoked activation in the R.DLPFC safely and effectively improves clinical symptoms and social dysfunction in MDD. Altered effective connectivity among the DLPFC, insula and ACC may provide mechanistic insights into its therapeutic effects. Trial Registration: chictr.org.cn; ChiCTR2300068273.

## Introduction

Major depressive disorder (MDD) is a debilitating mental illness characterized by persistent low mood and loss of interest, imposing significant socioeconomic and familial burdens [1]. A core yet under-addressed feature of MDD is social dysfunction, which is manifested by decreased social engagement (e.g., withdrawal from interpersonal activities), decreased social interaction frequency, and impaired relational quality [2, 3]. Social dysfunction persists beyond symptomatic remission and is now recognized as a critical predictor of the risk of disease recurrence and functional recovery [4, 5]. Despite its prognostic significance, targeted interventions for improving psychosocial function in MDD are currently lacking. Given this gap, developing treatment strategies targeting both mood symptoms and social dysfunction may yield enhanced therapeutic benefits.

Repetitive transcranial magnetic stimulation (rTMS) has been a well-established non-invasive treatment for MDD, typically targeting either the left or right dorsolateral prefrontal cortex (DLPFC). Beyond core mood symptoms, emerging evidence suggests that rTMS can also lead to meaningful improvements in psychosocial outcomes. For instance, rTMS has been shown to enhance self-reported social functioning and quality of life [6, 7]. Furthermore, rTMS may positively impact core social cognitive processes, such as improving emotion recognition abilities in MDD patients [8]. Compared to the left DLPFC (L.DLPFC) high-frequency (usually 5–20 Hz) stimulation, low-frequency (usually ≤1 Hz) stimulation targeting the right DLPFC (R.DLPFC) has been shown to produce comparable antidepressant effects and offer higher patient comfort and lower incidence of side effects [9–11]. On the other hand, right-sided stimulation has been found to impact social interactive behavior and its related brain activity in healthy participants [12–14]. The ultimatum game (UG) is a widely utilized paradigm for investigating social interactive behaviors. In the UG, a proposer suggests a way to divide a fixed sum of money. The responder has to accept or reject the proposal. If the responder accepts, the money is split as proposed; if rejected, neither player receives anything. Empirically, human responders consistently reject unfair offers (typically those below 20–30% of the total). The DLPFC is known to engage in fairness perception, cognitive control, and conflict resolution during UG [15, 16]. Consistent findings in healthy populations indicate that 1 Hz TMS of the R.DLPFC (but not L.DLPFC) enhances acceptance rates for unfair offers during this interactive economic game [12–14], suggesting a modulation of the balance between normative and economic motives. Additionally, neuroimaging results have demonstrated that R.DLPFC stimulation modulated functional activity in both the R.DLPFC and ventromedial prefrontal cortex, as well as their functional connectivity during the UG [12]. Utilizing this paradigm, altered social interactive behavior (lower acceptance rate) was observed in MDD [17–20] and behavioral parameter (learning rate) derived from computational modeling was found to be predictive of depression severity [21], which demonstrated that this paradigm can be utilized to objectively quantify social interactive behaviors and uncover underlying neuropsychological mechanisms. Currently, only one study investigated the effect of R.DLPFC TMS on social dysfunction in MDD patients by employing health-related quality of life scale and revealed that 1 Hz R.DLPFC stimulation improved social functioning in MDD patients [6]. Collectively, accumulated evidence implies that R.DLPFC stimulation may enhance clinical symptoms and social functioning in MDD, with UG task indices (acceptance rate and learning rate) serving as objective quantitative measures for assessing social function.

Recent studies have demonstrated that individualized magnetic stimulation targeting to the L.DLPFC based on resting-state functional connectivity [22, 23], or the visual region guided by task-evoked activation [24], significantly enhances therapeutic efficacy. However, the potential of precise individualized targeting of the R.DLPFC remains unexplored, particularly in the context of MDD. By leveraging task-evoked functional activation from the UG task, individualized R.DLPFC nodes, the key region determining social interactive behavior, can be mapped. This approach enables systematic investigation of the effect of individualized R.DLPFC stimulation on MDD.

Additionally, the neuromodulatory effect of R.DLPFC stimulation on brain functions remains unexplored in MDD, especially regarding stimulation-induced changes in interregional functional interactions during social interactions. Current research primarily focuses on the effects of TMS interventions on local brain activity or functional connectivity, with limited attention paid to effective connectivity. Notably, effective connectivity can provide directional information about interregional neural interactions, offering more precise characterization of brain functions [25]. We hypothesized that individualized stimulation of the R.DLPFC may induce targeted neuroplastic changes and alter effective connectivity between key brain regions involved in the UG task, leading to concurrent improvement in both depressive symptoms and social functioning in patients.

Currently, theta burst stimulation (TBS) is gaining growing interest due to its advantage of shorter treatment duration, with continuous TBS (cTBS) typically applied to the R.DLPFC and intermittent TBS (iTBS) to the L.DLPFC, particularly in accelerated protocols. Recently, Zhao et al. (2024) examined the clinical effects of accelerated schedules of iTBS and cTBS in MDD patients with moderate to severe suicidal ideation [26]. Treatments were delivered in 10 daily TBS sessions (1800 pulses/session) for 5 consecutive days. T1-weighted MRI data were utilized and neuronavigation was employed to target the group-average coordinates of the L.DLPFC and R.DLPFC based on studies by Fox et al. [27]. They found that accelerated schedules of L.DLPFC iTBS and R.DLPFC cTBS are comparably effective in managing antidepressant symptoms and that cTBS is potentially superior in reducing suicidal ideation and anxiety symptoms. However, previous meta-analysis of earlier randomized controlled trials of cTBS for treating MDD reported no advantage in treatment effects as compared to sham groups [28, 29]. We speculate that the discrepancies across studies may be related to stimulation dosage and TMS targeting methodology. Therefore, we anticipated that R.DLPFC stimulation with both accelerated cTBS pattern and individualized targeting method might be strong candidates for the treatment of depression and social functioning in MDD.

This study aims to evaluate the therapeutic efficacy of accelerated cTBS (1800 pulses/session across 30 sessions) targeting the individualized R.DLPFC, as identified by UG task-based functional localization, in patients with MDD. Specifically, we compared the effects of active versus sham cTBS stimulation on clinical symptoms and social function characterized by the UG task. We also examined the potential modulation effects of cTBS on effective connectivity and expected that there were different changes in effective connectivity after treatment between the active group and the sham group. At last, we further explored the link between effective connectivity changes and improvements in symptoms and social function. The findings may inform future clinical applications of individualized neuromodulation therapies in MDD.

## Methods and materials

### Study design

This study was preregistered at ChiCTR.org.cn prior to initiation (ChiCTR2300068273) and conducted at Beijing Anding Hospital, Capital Medical University in China. All procedures were performed in compliance with relevant laws and institutional guidelines. The study was approved by the hospital’s Institutional Review Board. The privacy rights of human subjects have been observed and all participants provided full written informed consent.

Participants were randomly allocated to one of two groups (active vs. sham cTBS). Randomization was performed by a researcher who was not involved in treatments. At baseline, clinical symptoms severity was measured using Hamilton Depression Rating Scale (HAMD-17), Hamilton Anxiety Rating Scale (HAMA), and Global Assessment of Functioning Scale (GAF) and MRI scanning was conducted. All patients underwent 10 days of cTBS (2 weeks, weekdays only). Post-treatment assessments were conducted within 3 days after treatment completion. The pipeline of the recruitment, allocation, treatment, and assessments of MDD are summarized in the Supplementary Methods (Fig. S1).

### Participants

All patients were recruited from Beijing Anding Hospital, Capital Medical University, and diagnosed with MDD utilizing the Mini International Neuropsychiatric Interview (M.I.N.I.) 7.0.2, a standardized and structured clinical assessment tool based on the DSM-5 criteria. They were outpatients, aged 18-45 years, right-handed, educated at least junior high school, and had received no antidepressants and antipsychotics for at least 2 weeks before treatment. Additionally, patients that had a HAMD-17 score ≥14 were included. Exclusion criteria are provided in the Supplementary Methods, Exclusion criteria of participants.

From April 2023 to March 2024, a total of 215 patients were screened, with 70 randomized assigned to either the active group (*n* = 37) or the sham group (*n* = 33). To be noted, none of the enrolled participants met the criteria for treatment-resistant depression. The sample size calculation is detailed in the Supplementary Methods. Of these, 63 patients (active group, *n* = 33; sham group, *n* = 30) completed intervention, clinical assessments, and MRI scans (Fig. S1). Baseline demographics and clinical characteristics were compared between groups (Table 1).

**Table 1:** Demographic and clinical characteristics of the active and sham cTBS groups.

Characteristics	Active group (*n* = 37)	Sham group (*n* = 33)	*t*/χ^2^	*p*
Age(years)	31.16 ± 7.12	31.45 ± 7.46	–0.17^a^	0.867
Sex(male/female)	15/22	18/15	1.37^b^	0.241
Education status			0.14^b^	0.934
high school/college	3	3		
undergraduate	24	20		
postgraduate or above	10	10		
Illness duration (weeks)	96.30 ± 167.73	61.85 ± 85.22	1.06^a^	0.291
Type of episode (first episode/recurrent)	14/23	12/21	0.02^b^	0.899
HAMD-17 (baseline)	22.92 ± 5.78	23.15 ± 3.83	–0.20^a^	0.845
HAMA (baseline)	21.46 ± 8.77	20.00 ± 5.53	0.82^a^	0.414
GAF (baseline)	82.24 ± 3.30	82.96 ± 1.77	–1.11^a^	0.270
Acceptance rate (baseline)	0.41 ± 0.38	0.55 ± 0.32	–1.58^a^	0.120
Learning rate (baseline)	0.06 ± 0.04	0.20 ± 0.08	–7.28^a^	< 0.001

^a^Two-sample *t* test.

^b^Chi-squared test.

*HAMD-17* 17-item Hamilton Depression Rating Scale, *HAMA* Hamilton Anxiety Rating Scale, *GAF* Global Assessment of Functioning Scale.

### Randomization and blinding procedures

An independent research assistant generated the randomization code (A or B) using a computer program. This code was then utilized to assign the correct labeled coil for each patient. During the entire treatment, participants were instructed not to discuss intervention details with each other. Participants, outcome assessors, therapists, and TMS operators were blind to group allocation. After the stimulation, we also conducted the blinding evaluation through a 0 (100% confirmed sham stimulation) - 10 (100% confirmed active stimulation) likert scale for all the patients. The blinding evaluation scores in the active group (6.06 ± 1.94) and the sham group (5.30 ± 2.31) did not differ significantly (*t* = 1.42, *p* = 0.160).

### MRI data acquisition

**Fig. 1:**
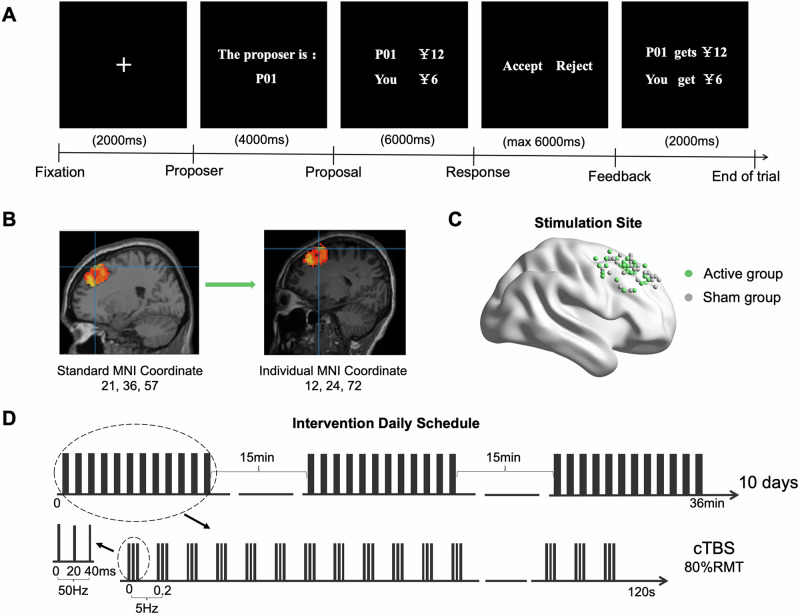
Ultimatum Game paradigm, localization of the individualized target, and cTBS protocol and pattern illustration. **A** Flowchart of the UG task; **B** spatial normalization of task-based peak activation from standard to native space in a representative participant; **C** the individualized target coordinates of each patient in standard MNI space; **D** cTBS treatment schedule and schematic of the cTBS pattern. MNI Montreal Neurological Institute, RMT resting motor threshold, cTBS continuous theta burst stimulation.

The MRI data, including T1-weighted structural, resting-state fMRI, UG task-based fMRI data, were collected before and after treatment using a Siemens Prisma 3.0 T MRI scanner at Beijing Anding Hospital. Resting-state fMRI data were not used in the present study. Scanning parameters are detailed in the Supplementary Methods, MRI data acquisition. For task-based fMRI (Fig. 1A), we employed an UG paradigm comprising 6 fair and 18 unfair offers, consistent with our prior studies [17, 20, 21]. Full paradigm details appear in the Supplementary Methods, UG protocol.

### Personalized target identification

Personalized targets were generated for each patient using baseline task-fMRI scans. The process involved manual coregistration, fMRI preprocessing, individual T1 segmentation, definition of R.DLPFC regions, and calculation of brain activation to identify the voxel with the strongest activation when facing unfair offers compared with fair offers as the personalized stimulation target (Fig. 1B). The final individualized target coordinates of each patient in MNI standard space are shown in Fig. 1C. Additional details are provided in the Supplementary Methods, Personalized target identification.

### cTBS protocol

We used the Magstim rapid2 stimulation equipment with two figure-of-eight-shaped coils (labeled A and B for active and sham conditions, respectively). The sham coil has identical appearances and produce identical stimulation sounds with the real coil but without real magnetic stimulation. The system also incorporates a real-time neuronavigation system (ANT neuro, visor 2) to precisely track the target during stimulation, guiding the placement of the coil. Based on a simulated electrical field model integrated within the navigation platform, coil orientation was individually optimized by selecting the configuration that yielded the highest normalized electric field strength at the target coordinates. cTBS was delivered at 80% resting motor threshold on the R.DLPFC. Each session of the cTBS involves a 120 s stimulation of three 50-Hz bursts at 5 Hz, for 1800 pulses total. Three sessions (5400 pulses/day) were given daily, separated by 15-minute intervals. All patients underwent 10 days of cTBS over a 2-week period (Fig. 1D). Additional details are provided in the Supplementary Methods, cTBS procedures.

### Clinical and behavioral outcomes

The primary outcome was the score changes from baseline to the end of stimulation in the HAMD-17. Secondary outcomes included the score changes from baseline in the HAMA, GAF, and social interactive indices (including acceptance rate of offers and learning rate as described below) in the UG task. Secondary outcomes also included the response rate and remission rate of the patients according to the HAMD-17 and HAMA. Response was defined as a reduction ≥50% and remission was defined as a score ≤7 after 2 weeks of intervention. Adverse events were monitored and recorded throughout the intervention period.

### Estimation of learning rates

A reinforcement learning model was utilized to depict the learning processes during the UG in both the active and sham groups. Building upon our prior research [21, 30, 31], the hierarchical Bayesian estimator was used, which enabled a simultaneous estimation of individual and group-level learning parameters, one focal free parameter is the learning rate α, which captures how fast one learns and adjusts one’s norm. When α is large, individuals can rapidly adjust their fairness norms to adapt to new environments; whereas when α is small, individuals respond more slowly to new information and tend to maintain their original fairness perceptions (see Supplementary Methods, Estimation of learning rates). In addition, the learning rate (α) has been shown to be clinically meaningful. Our previous longitudinal twin study where participants acted as responders in the UG during early adulthood, individual differences in learning rates significantly predicted the severity of depressive symptoms measured eight years later [21]. This underscores the role of adaptive social learning in long-term mental health outcomes, particularly in the context of MDD.

### Effective connectivity analyses

A standard GLM analysis was firstly conducted for the task-based neuroimaging data to identify brain regions exhibiting activation modulation by the group × time interaction during the offer presentation phase. Seven functionally significant regions based on this analysis were selected as volumes of interest for subsequent effective connectivity analysis (Table S1). We then employed determined dynamic causal modeling (DCM) to examine directional connectivity patterns among these regions. At the group level, we implemented parametric empirical Bayes (PEB) for hierarchical parameter estimation and Bayesian model reduction (BMR) for model comparison to estimate the group mean and the effects of group, time and group × time interaction for average connectivity (matrix A) and task modulation parameters (matrix B), separately. The detailed analytical procedure can be seen in the Supplementary Methods, Effective connectivity analyses.

### Statistical analyses

Statistical analyses were performed using SPSS22.0 and R4.4.0. Primary and secondary outcomes were analyzed using an intention-to-treat (ITT) approach with general liner mixed model (GLMM) to examine the group, time, and group × time interaction effects. If a significant group × time interaction effect was observed, post-hoc analysis was conducted utilizing Bonferroni correction and 5% significance level (two-tailed). For the response rate and remission rate, ITT analysis was also conducted. Additionally, the learning rate estimates were compared between the baseline and post-treatment time points to determine the treatment effect in learning processes for each group. The posterior means and 89% highest density interval (HDI) were reported as summary statistics. For exploratory correlation analyses between improvements in depressive, anxiety, and GAF scores, as well as between changes in identified effective connectivity (post-treatment minus pre-treatment) and clinical/behavioral improvement, Pearson correlation coefficients were calculated within each group. The significance level was set at 5% (two-tailed).

## Results

### cTBS-induced improvements in depressive and anxiety symptoms and general social functioning

**Fig. 2:**
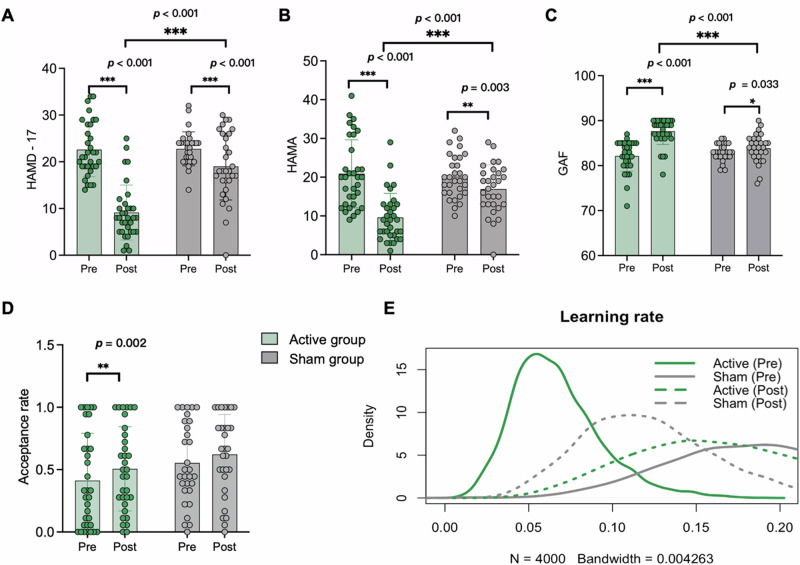
cTBS-induced changes in clinical symptoms and social functioning. Changes in **A** depressive symptoms, **B** anxiety symptoms, and **C** general social functioning pre- and post-treatment in each group. **D** Changes in acceptance rates pre- and post-treatment in each group. The significance markers are based on paired-sample *t-*tests, as a GLMM revealed no significant main effect of group or group-by-time interaction for this measure. **E** Changes in learning rates pre- and post-treatment in each group. HAMD-17 17-item Hamilton Depression Rating Scale, HAMA Hamilton Anxiety Rating Scale, GAF Global Assessment of Functioning Scale.

A significant group × time interaction effect was found for depressive symptoms (HAMD-17 scale) (*F* = 23.12, *p* < 0.001) (Fig. 2A), along with main effects of time (*F* = 80.29, *p* < 0.001) and group (*F* = 25.41*, p* < 0.001). Post-hoc analysis revealed a greater reduction in HAMD-17 in the active group compared to baseline (mean change 13.76, *p* < 0.001), with a smaller but also significant reduction in the sham group (mean change 4.15, *p* < 0.001). At week 2, the active group showed significantly lower HAMD-17 scores than the sham group (mean difference 9.84, *p* < 0.001). Clinical response rate (active *n* = 24/37, 64.9%, sham *n* = 2/33, 6.1%) and remission rate (active *n* = 14/37, 37.8%, sham *n* = 2/33, 6.1%) for depressive symptoms were significantly higher in the active group (all *ps* < 0.01, Table 2).

**Table 2:** Response rate and remission rate for depressive and anxiety symptoms in the intention-to-treat analysis.

	Active group (*n* = 37)	Sham group (*n* = 33)	χ^2^	*p*
HAMD-17				
Response Rate (n/%)	24 (64.9)	2 (6.1)	31.63	< 0.001
Remission Rate (n/%)	14 (37.8)	2 (6.1)	9.38	0.002
HAMA				
Response Rate (n/%)	22 (59.5)	4 (12.1)	16.74	< 0.001
Remission Rate (n/%)	15 (40.5)	1 (3.0)	13.92	< 0.001

Similarly, for anxiety symptoms (HAMA scale), there was a significant interaction effect (*F* = 14.98, *p* < 0.001) (Fig. 2B), along with main effects of time (*F* = 42.08; *p* < 0.001) and group (*F* = 6.89; *p* = 0.010). Post-hoc analysis showed a greater reduction in the active group compared to baseline (mean change 12.13, *p* < 0.001), while the sham group also showed a smaller but significant reduction (mean change 3.07, *p* = 0.003). At week 2, HAMA scores were significantly lower in the active group compared to the sham group (mean difference 7.33, *p* < 0.001). Clinical response rate (active *n* = 22/37, 59.5%, sham *n* = 4/33, 12.1%) and remission rate (active *n* = 15/37, 40.5%, sham *n* = 1/33, 3.0%) for anxiety symptoms were also significantly higher in the active group (all *ps* < 0.001, Table 2).

For general social functioning (GAF scale), a significant interaction effect was also observed (*F* = 20.08, *p* = 0.003) (Fig. 2C), along with main effects of time (*F* = 41.38, *p* < 0.001) and group (*F* = 9.08, *p* < 0.001). Post-hoc analysis showed a greater increase in GAF scores in the active group (mean change 5.40, *p* < 0.001) compared to a smaller but still significant increase in the sham group (mean change 0.97, *p* = 0.033). At week 2, GAF scores were significantly higher in the active group than in the sham group (mean difference 3.70, *p* < 0.001).

Additionally, correlation analyses were performed to examine the relationships among improvements in depressive symptoms, anxiety symptoms, and general social functioning. In the active group, changes in HAMD-17 and HAMA scores were strongly correlated (*r* = 0.794, *p* < 0.001), and changes in HAMD-17 and GAF scores were also significantly correlated (*r* = 0.404, *p* = 0.020). Although a positive association was observed between HAMA and GAF improvements, it did not reach statistical significance (*r* = 0.260, *p* = 0.144). In the sham group, all three correlations were statistically significant (all *ps* < 0.001).

### cTBS-induced changes in social interactive indices

For the acceptance rate to offers in the UG, significant main effect of time was observed (*F* = 12.24, *p* = 0.001), indicating increased acceptance following treatment. No significant group effect (*F* = 2.07, *p* = 0.155) or interaction effect (*F* = 0.37, *p* = 0.544) was observed (Fig. 2D). Paired-sample *t*-tests were conducted to examine within-group effects and revealed a significant increase in the active group (*t* = –3.40, *p* = 0.002), but not in the sham group (*t* = –1.78, *p* = 0.086).

As to the learning rate during the social interactive process, density map revealed a significant group × time interaction effect in the UG task (89% HDI: [0.02, 0.35]). Pre- and post-treatment comparisons showed a significant increase in the active group (89% HDI: [0.01, 0.21]), whereas the sham group exhibited a non-significant decrease (89% HDI: [-0.21, 0.05]) (Fig. 2E).

### Adverse effects

No severe adverse effects occurred during the experiment. The most common adverse effect was localized pain at the stimulation target, which occurred only in the active group (12%). Statistical analysis revealed a significant difference of localized pain between the active and sham groups (*χ*^*2*^ = 3.88, *p* = 0.049). However, the pain gradually subsided shortly after stimulation and completely disappeared by the end of the session. No other adverse effects showed any significant differences between the two groups (Table S2).

### cTBS-induced changes in brain activation during the UG

**Fig. 3:**
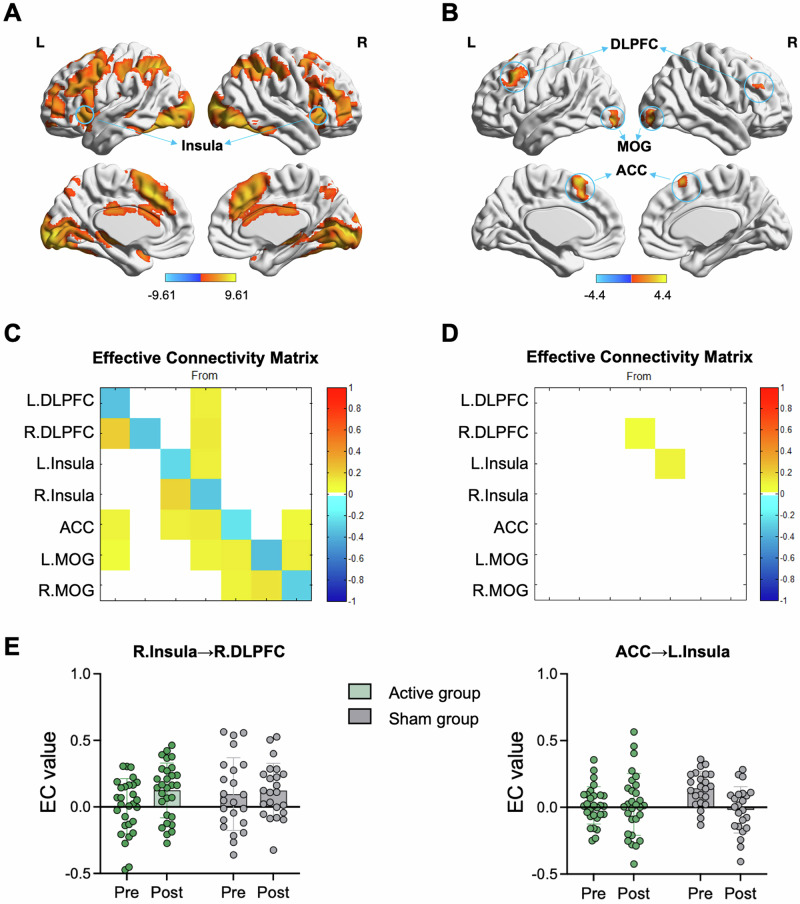
cTBS-induced changes in brain activation and effective connectivity during the UG. **A** The regions showing brain activity during the offer presentation stage (voxel-level *p* = 0.001, cluster-level FWE *p* < 0.05). **B** The regions showing brain activity modulated by group × time interaction for the interested [unfair-fair] contrasts (voxel-level *p* = 0.005, >30 voxels). These results did not survive multiple comparisons correction (cluster-level FWE *p* < 0.05) and should be considered exploratory. **C** The group mean effective connectivity across all subjects and conditions (*Pp* > 0.95). These parameters represent the average connectivity during the offer presentation. Overall, the regions showed excitatory influences between each other (yellow blocks) and the self-connections of each region were inhibitory (blue blocks). **D** The connections, i.e., R.Insula→R.DLPFC and ACC→L.Insula, modulated by group × time effects (*Pp* > 0.95). **E** The effective connectivity strengths identified in the interaction effect were plotted in each group and time point. L left, R right, EC effective connectivity, FWE family wise error, Pp posterior probability.

During the offer presentation stage, widespread brain areas including the anterior cingulate cortex (ACC), the bilateral DLPFC, the bilateral insula, and the visual cortex, were activated across participants (cluster-level FWE *p* < 0.05; Fig. 3A). Within this brain mask, we found that the brain activity in the ACC, bilateral DLPFC and bilateral middle occipital gyrus (MOG) for the [unfair-fair] contrasts was modulated by group × time interaction in a less strict threshold (voxel-level *p* = 0.005, >30 voxels; Fig. 3B), although these brain regions failed in passing multiple comparisons (cluster-level FWE *p* < 0.05). Considering the insular’s important role in the interactive process of the UG task [15], the 5 regions identified in the group × time interaction effect and the bilateral insula activated in the offer stage were used to build a model to investigate effective connectivity while participants were facing offers (Table S1).

### cTBS-induced changes in effective connectivity

Across all the participants, we found task independent effects on the connections across conditions (i.e., matrix A) but no task modulation effects on the connections (i.e., matrix B).

Using DCM/PEB analysis, we found strong evidence to support that there were effective connections among the bilateral DLPFC, insula, MOG and ACC across the task conditions (*Pp* > 0.95; Fig. 3C). Overall, the between-region effective connections were excitatory and the self-connections in these regions were inhibitory and strong. The connections showing group or time effect can be seen in Supplementary Results (Fig. S2 and S3).

Figure 3D shows group × time interaction effect in connectivity. Connections of the R.Insula→R.DLPFC and the ACC→L.Insula were modulated by group × time interaction. Specifically, the R.Insula→R.DLPFC connectivity in the active group was increased after treatment while it stayed unchanged in the sham group; the ACC→L.Insula connectivity stayed unchanged in the active group while it was decreased in the sham group after treatment (Fig. 3E).

We further examined correlations between the effective connectivity changes (post-treatment minus pre-treatment) and clinical/behavioral improvement of the patients. Exploratory correlation analyses did not reveal any statistically significant associations within either treatment group (Table S3).

## Discussion

This study is the first to investigate the efficacy of accelerated cTBS with an individualized targeting method in MDD. Following 10 days cTBS stimulation, our results demonstrated significant improvements in clinical symptoms, general social functioning, and social interactive indices compared to a sham intervention. These findings provide strong evidence supporting the therapeutic potential of individualized cTBS in multiple domains, while also highlighting its good tolerability and safety. Moreover, neuroimaging revealed altered effective connectivity among the DLPFC, insula, and ACC induced by cTBS stimulation.

The significant improvement in clinical scales observed in the active group was consistent with previous studies showing that stimulating the R.DLPFC could effectively alleviate both the depressive and anxiety symptoms [10, 26, 32] and improve general social functioning in patients with MDD [6]. Notably, our sham control group also exhibited significant improvements in both clinical symptoms and general social functioning. Several factors may contribute to the placebo effects, such as the non-specific therapeutic effects of increased clinical attention and patient expectations, and potential natural symptom fluctuation during the study period. Although the improvement was also observed in the sham group, the active group demonstrated substantially greater improvement. Furthermore, the correlation analyses revealed significant interrelationships among improvements in depressive symptoms, anxiety symptoms, and general social functioning. These findings underscore the interconnected nature of symptom recovery across affective and functional domains. Together, these findings support the clinically meaningful efficacy of an accelerated cTBS protocol with personalized targeting, as reflected in significant and broad symptom reduction and functional gains over the two-week treatment period.

The response rate (64.9%) and remission rate (37.8%) following 2 weeks stimulation observed in our study were relatively higher compared with conventional FDA-approved protocol (47% and 27%) which requires 4–6 weeks of treatment [33]. This encouraging clinical efficacy may be attributed to the individualized precision of stimulation targets and the adequate number of treatment sessions. First, extensive research has confirmed that neuronavigated individualized targeting demonstrates superior antidepressant efficacy compared to conventional localization methods [34]. Approximately one-third conventional targets deviate from the intended stimulation site [35]. In contrast, neuronavigation-guided individualized targeting enables precise stimulation of the target brain region and reported superior outcomes compared to non-individualized control groups [36]. Secondly, our study employed an intensive treatment protocol of 30 sessions within two weeks, which may contribute to better clinical outcomes. Previous studies found that greater numbers of TMS sessions were associated with more significant improvements in depressive symptoms [37] and accumulating evidence suggests that approximately 30 sessions are required to achieve optimal therapeutic effects [38]. The dose-dependent treatment effects may be mediated by neuroplasticity mechanisms - repeated stimulation sessions may facilitate the transformation of short-term neuroplastic changes into long-term functional brain reorganization [39], ultimately manifesting as improved clinical outcomes.

At the behavioral level, we observed a pattern wherein the acceptance rates for unfair offers increased from pre- to post-treatment in the active cTBS group, while no such change was evident in the sham group. However, it should be noted that this difference in change over time did not constitute a statistically significant group-by-time interaction. This finding is supported by rTMS studies in healthy populations, where R.DLPFC stimulation similarly increased acceptance rates in the UG [13, 14], mirroring our observations in MDD patients. In addition, the active group exhibited a significant improvement in learning rate, while the sham group showed a decline. The change in the learning rate in the active group may be related to the positive impact of TMS on cognitive networks, especially in enhancing executive functions and learning abilities [28, 40–42] and allowing individuals to adjust cognitive strategies more rapidly when processing new information [41]. Previous research has identified deficits in fairness norm adaptation among depressed patients during the social interactive task [42]. Our recent study revealed a significant correlation between learning rate and depressive symptoms, demonstrating that learning rate can predict the severity of depressive symptoms eight years later [21]. An improved learning rate in the current study further suggests that after TMS stimulation, depressed individuals exhibit heightened sensitivity to social cues, more responsive adaptation to new social information, and better adjustment to changing rules, although the interpretation of this result is constrained by the pre-existing baseline difference between the groups. In contrast, the decline in learning rates in the sham group may results from the lack of real stimulation, leading to persistent or even increased cognitive load, which negatively affected their social cognitive performance.

Notably, our findings also reveal important insights into neural mechanisms of personalized cTBS treatment in MDD. We firstly found that the brain activations of the bilateral DLPFC, bilateral MOG, and ACC during the task were modulated differently by treatment in the active and sham groups. All these regions were key brain regions during social processing involved in the task [43]. However, it is important to note that these effects were observed under a less strict statistical threshold and did not survive multiple comparisons correction; therefore, they must be interpreted as exploratory. Based on these exploratory findings and by using DCM analysis, we further found that effective connections of the R.Insula→R.DLPFC and ACC→L.insula were modulated by group × time interaction. Specifically, the R.Insula→R.DLPFC connectivity in the cTBS group was increased after treatment but remained unchanged in the sham group. The right insula serves as a core hub of the salience network (SN), while the R.DLPFC represents a central node of the executive control network (ECN) [44]. This finding demonstrates that R.DLPFC-targeted TMS stimulation dynamically modulates cross-network communication between SN and ECN, suggesting TMS induces network-level effects beyond local stimulation targets. Previous studies have found that rejected offers, compared to accepted ones, elicit stronger activation in brain regions such as the R.DLPFC [45]. In our study, cTBS targeting R.DLPFC enhanced the effective connectivity from the right insula to the R.DLPFC. These findings suggest that cTBS may modulate the emotion-cognition integration circuit, potentially facilitating a more balanced neural processing of fairness-related decisions.

Meanwhile, the ACC→L.insula connectivity remained stable in the cTBS group but exhibited significant reduction in the sham group after treatment. Both the insula and ACC are core components of the salience network. The dorsal ACC, as another principal node of this network, plays pivotal roles in conflict monitoring and emotion regulation [46]. Previous studies have demonstrated reduced ACC activation during conflict tasks [47] and diminished ACC-insula functional connectivity in MDD patients [48]. Our finding suggests that cTBS may exert therapeutic effects by maintaining network integration balance within the SN. This stabilization of SN connectivity patterns potentially represents a novel mechanism underlying clinical improvements. However, the lack of healthy controls in the current study prevents conclusive determination of whether abnormal effective connectivity exists in MDD patients. Furthermore, the lack of significant brain–behavior associations within each treatment group may be attributable to limited sample size or may suggest that clinical efficacy is not mediated by simple linear changes in individual connections, but rather through broader network-level reorganization or heterogeneous response pathways across patients.

### Strengths and limitations

This study represents the first investigation employing individualized cTBS targets to examine effects on both clinical symptoms and social dysfunction in MDD. As a single-center study with 70 MDD participants, our research benefits from a relatively large sample size while maintaining strong patient homogeneity. Our neuroimaging-based, task-guided personalized targeting approach revealed a novel spatial distribution pattern of stimulation targets that differs substantially from conventional targeting methods. Furthermore, the study demonstrated excellent retention, with only a 10% attrition rate at treatment endpoints—significantly lower than the typically anticipated 20% dropout rate in similar clinical trials.

Several limitations of this study should be acknowledged. First, although our sample size (*n* = 70) is relatively large for a neuromodulation study, the single-center design may limit generalizability. And the sample was characterized by relatively mild baseline depressive severity and an absence of treatment resistance, which may also impact the broader applicability of the findings and necessitates validation through future multi-center replication in more severe and treatment-resistant populations. Second, the assessment of outcomes was conducted over a relatively short, two-week period following treatment. This restricts our ability to evaluate the long-term efficacy and sustainability of the clinical and neurophysiological improvements observed. Future studies with extended follow-up durations (e.g., 1 to 3 months or longer) are necessary to determine the durability of the treatment effects and to monitor potential relapse. Third, we did not account for clinical subtypes of MDD in our study. Consequently, our study precludes definitive conclusions on whether the treatment efficacy differed across patient subgroups, underscoring the need for future research to incorporate these diagnostic specifiers to elucidate potential moderating effects and better account for the clinical heterogeneity of MDD. Fourth, although the GAF is a well-established global measure, its breadth means it may not fully capture specific aspects of social functioning relevant to TMS treatment. The absence of more specialized social functioning measures is a limitation, and future studies would benefit from incorporating such tools to provide a more nuanced understanding. Another methodological limitation is the inherent coupling between functional targeting and electric field optimization in our study design. While we maximized the overlap between the simulated electric field peak and the functionally derived target, we cannot definitively attribute the observed effects to either component alone. Future studies with factorial designs are needed to parse their unique contributions. Furthermore, the spatial concordance between the peak of the simulated electric field and the functionally defined target coordinate was evaluated mainly via visual inspection. Future technical work would benefit from implementing formal volumetric overlap metrics (e.g., Dice coefficient) between the e-field distribution and the target region. In addition, while this study employed both an accelerated cTBS protocol and individualized targeting, the respective contributions of these two factors to the observed clinical efficacy remain unclear. Our design does not allow for firm conclusions about the specific advantage of individualized targeting compared to standard localization methods. Future research should systematically compare accelerated versus standard protocols, with and without personalized targeting, to disentangle their unique therapeutic mechanisms. Moreover, while recent large-scale observational studies have found minimal clinical benefit for targeting the classical subgenual anterior cingulate cortex (sgACC)-DLPFC circuit [49–51], definitive conclusions regarding personalized versus standard TMS targeting await future, large-scale randomized trials.

## Conclusion

This study is the first to apply individualized, accelerated cTBS targeting the R.DLPFC, based on task-evoked brain activation. We demonstrated that R.DLPFC-targeted accelerated cTBS significantly alleviates depressive and anxiety symptoms while enhancing social functioning, social interactive behavior, and learning rates compared to a sham intervention. These improvements may stem from changes in functional interactions among the DLPFC, insula, and ACC induced by TMS. Notably, the intervention was well-tolerated, with no major adverse effects, supporting its favorable safety profile. Our findings suggest that this precision-targeted accelerated cTBS approach could represent a promising therapeutic strategy for addressing both core clinical symptoms and functional impairments in MDD.

## Supplementary information

Supplementary Information

## Data Availability

Data is available on request from the corresponding authors.
