# Supplementary material for: Task-guided accelerated cTBS simultaneously treats depression and social dysfunction in patients with major depressive disorder: a randomized clinical trial
Source: Neuropsychopharmacology. 2026 Feb 20;51(7):1290–9. doi: 10.1038/s41386-026-02365-7 (PMC13213039; doi:10.1038/s41386-026-02365-7)
Supplement: Supplementary file 1 — Supplementary Information [file 41386_2026_2365_MOESM1_ESM.docx]

SUPPLEMENTARY INFORMATION

**Task-Guided Accelerated cTBS Simultaneously Treats Depression and Social Dysfunction in Patients with Major Depressive Disorder: A Randomized Clinical Trial**

Jin *et al.*

**This file includes:**

I. Supplementary Methods: pages 2-11

Figures S1

II. Supplementary Results: pages 12-13

Figures S2 to S3

III. Supplementary Discussion: pages 14-15

Tables S1 to S3: page 16-17

IV. Supplementary References: pages 18-19

1. **Supplementary Methods**

###
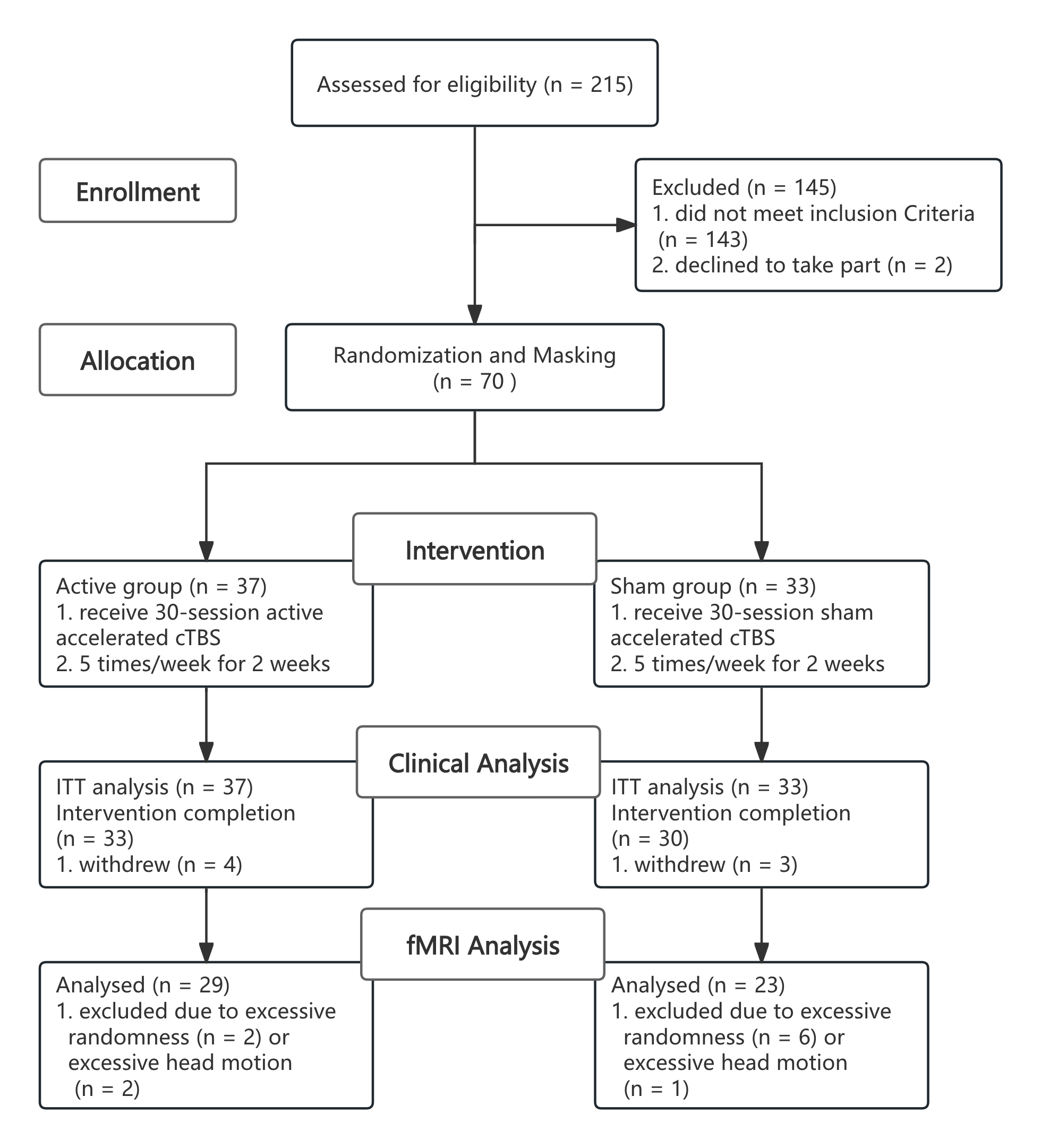


### Figure S1. Flowchart of patient’s inclusion. ITT, intention-to-treat.

### Exclusion criteria of participants

The exclusion criteria included: (1) other diagnoses such as schizophrenia, alcohol and drug dependence that meet the DSM-5 diagnostic criteria; (2) history of seizure disorder, trauma resulting in loss of consciousness, history of major neurological or physical disorders that could lead to an altered mental state; (3) history of mania or hypomania; (4) MDD with psychotic symptoms; (5) current pregnancy or breastfeeding; (6) history of prior treatment with physical therapies such as MECT/ECT, TMS, or tDCS within the past 6 months; (7) contraindications to MRI.

**Sample size calculation**

The sample size (N=70) for this study was determined based on a comprehensive consideration of the following three aspects.

(1) Sample size required for an RCT study involving rTMS intervention

With reference to previous meta-analyses employing RCT designs to investigate the effects of rTMS intervention in depression - Martin et al. (2017) and Iimoria et al. (2019) (1,2) - the average sample sizes per group in the included studies were 15.6 and 17.1, respectively. Among these, four studies with larger sample sizes (≥20 participants per group) reported average sample sizes of 27.5 and 27 per group.

(2) Sample size required for task-based neuroimaging research

Based on previous neuroimaging studies, the sample sizes included in each group were referenced from meta-analyses on UG fMRI studies in healthy populations by Gabay et al. (2014) and Feng et al. (2015) (3,4), which reported average sample sizes of 25.6 and 27.1 per group, respectively.

(3) We used G*Power software to estimate the sample size.

Assuming a Cohen's d of 0.8, with 80% power to detect an effect at a two-sided alpha level of 0.05, the calculated required sample size was 26 cases per group.

Based on the comprehensive considerations above, this study aims to include 28 participants per group after the intervention. Accounting for a 20% attrition rate commonly observed in longitudinal observational studies, the target sample size results in a total planned enrollment of 70 patients.

### Concomitant therapies

During the trial, concomitant use of antidepressants, anxiolytics, antipsychotics, or mood stabilizers was strictly prohibited throughout the TMS treatment period. For cases of severe insomnia, short-term use of benzodiazepines or non-benzodiazepine hypnotics was permitted for a maximum duration of 7 consecutive days, with the requirement that all such medications be discontinued at least 8 hours prior to any clinical assessments. Additionally, all participants were required to abstain from receiving any physical therapy or psychotherapy outside the scope of this study during the 2-week TMS treatment course.

### MRI Data Acquisition

The T1-weighted structural scans were conducted using the following parameters: TR = 2530 ms, TE = 4.21 ms, FOV = 256 × 256mm, FA = 7°, slice thickness = 1mm. The resting-state fMRI scans and task-based fMRI data were collected using the following parameters: TR = 2000 ms, TE = 30 ms, FOV = 224 × 224 mm, FA = 90°, slice thickness = 3.5mm, 33 slices, no gap. Stimuli were presented with E-prime 2.0 software (Psychology Software Tools, Pittsburgh, PA, USA) on a personal computer, back-projected onto a screen using a liquid crystal display projector and viewed by the participants through a mirror mounted on the MRI head coil. The scanner was triggered by a signal generated by E-prime stimulus presentation software to synchronize each volume acquisition with the onset of a visual stimulus.

### UG protocol

The Ultimatum Game (UG) paradigm was adopted from our previous studies (5–7). Participants played a one-shot anonymous UG while undergoing the task-based fMRI scanning. UG offers unparalleled ecological validity compared to traditional social cognition experiments by simulating interactive decision-making scenarios (6) and allowing for a quantitative analysis of decision-making behavior within social interactions (8). In the UG paradigm, two participants were engaged in dividing a fixed sum of money. One participant, the proposer, determines how to allocate the amount, while the other, the responder, can either accept or reject the offer. If accepted, the money is divided according to the offer; if rejected, both participants receive nothing (9).

Before scanning, MDD patients received detailed instructions about the UG rules and completed a series of test questions to ensure full comprehension. During the scan, MDD patients played as responders and were required to accept or reject the allocation by pressing a button during decision phase. They were told that the proposals they viewed had been submitted by previous participants and that each trial involved a different, anonymous partner (one-shot design). In addition, to enhance ecological validity and participant engagement, they were told that we would also submit their proposals after the experiment and that their proposals might be used in future game sessions with other participants. In reality, the participants’ proposals were not used beyond their function as a cover story. Furthermore, to minimize uncontrolled social or emotional cues, all proposers were represented by alphanumerical codes rather than photographs or real names. The UG paradigm included 24 trials, all presented in random order. Each trial consisted of the following steps: (1) Preparation phase, (2) Presentation of the proposer’s ID, (3) Presentation of the allocation scheme, (4) Participant’s decision phase, and (5) Outcome presentation phase (Figure 1A). To control for the influence of the allocated amount on fairness perception, the responder’s allocation amount was fixed at 9, 10, or 11 yuan, with eight allocation schemes for each condition. The fairness level of the schemes ranged from 50% to 4%, and all three responder allocation conditions were evenly distributed across this range. Referring to previous research (10), schemes with a fairness level between 50% and 38% (6 schemes) were defined as fair offers, while those between 38% and 4% (18 schemes) were defined as unfair offers. We also implemented a random payment method in our experiment. Participants were informed that, after the task, they would be paid in cash based on two randomly chosen offers out of all the proposals.

### Personalized target identification

The individualized brain activation maps for depressed patients were obtained using task-based fMRI from the UG paradigm during the baseline fMRI scan. The coordinates with the strongest activation within the right DLPFC area when facing unfair offers compared with fair offers were identified as the individualized rTMS treatment target. Specifically, task-based fMRI image preprocessing was performed using DPABI_V6.1 (https://rfmri.org/DPABI) running on a Matlab R2014a platform (MathWorks, Natick, MA). The preprocessing included slice time correction, realignment, normalization, and resampling to 3 × 3 × 3 mm^3^, and smoothing using a 6-mm full-width-at-half-maximum Gaussian kernel. Then, a GLM analysis was performed using SPM12 to identify brain activation regions under the contrast of [unfair - fair] offers for each participant. A right DLPFC mask was used and created as the mirrored brain region corresponding to the left DLPFC mask defined in the previous studies (11). Specifically, 20 mm radius spheres centred at the mirrored coordinate of the left Brodmann area 9 (BA9) (MNI: -36, 39, 43), BA46 (MNI -44, 40, 29), the “5-cm” TMS site (MNI: -41, 16, 54), and group average stimulation target (MNI: -37, 26, 49) were combined with the grey matter mask in DPABI. The coordinates with the strongest activation within the right DLPFC mask were detected in the standard space first, and then converted to the individual space, which served as the individualized TMS treatment target (Figure 1B). The final individualized target coordinates of each patient in MNI standard space are shown in Figure 1C.

### cTBS procedures

Structural and functional MRI scans were obtained from all participants before intervention to generate the personalized stimulation target for each patient. Participants were randomly assigned to one of the two groups after identifying personalized stimulation targets. We used the Magstim rapid 2 stimulation equipment with two figure-of-eight-shaped coils delivering active or sham stimulation, respectively. Importantly, both the coils have identical appearances and produce identical stimulation sounds. To maintain blinding among the TMS operators, no indications of the active or sham coil were provided and just a number code was labeled for each coil. An unblinded research assistant assigned the correct coil number code for each patient based on their group allocation before treatment. The system also incorporates a real-time neuronavigation system (ANT neuro, visor 2) to precisely track the target during stimulation, guiding the placement of the coil. The TMS coil was positioned tangentially to the scalp at the predefined stimulation site, with the handle initially pointing posterolaterally at approximately 45° to the mid-sagittal plane. Starting from this initial orientation, the coil was rotated around the rotation axis of the stimulation site. During TMS neuronavigation, as the coil was moved over the patient's head, the system projected the estimated induced electric field in real time onto the displayed curvilinear anatomical slice. The coil orientation used during stimulation was determined by selecting the configuration that yielded the highest normalized electric field strength at the target coordinates in simulation. Based on the participant's individual anatomical MRI and the defined stimulation target, the system provided a visual guidance interface showing the acceptable range for coil position and orientation. Throughout the stimulation session, coil position and orientation were continuously monitored and maintained within this system-defined acceptable range to ensure accurate and individualized stimulation delivery.

Resting motor threshold (RMT) was determined at the contralesional primary motor cortex as the minimum intensity required to elicit first dorsal interosseous muscle contractions in more than half of ten trials. Active or sham cTBS was delivered at 80% RMT on the right DLPFC. Each session of the cTBS involves a 120 s stimulation of three 50-Hz bursts at 5 Hz. All patients underwent 10 days of cTBS, with 3 sessions per day and a 15-min interval between two adjacent sessions. Therefore, the total number of stimuli was 54,000 (1,800 pulses/session, 5,400 pulses/day, 54,000 in 10 days) (Figure 1D). The sham group followed the same protocol as the active group, but without real magnetic stimulation—only the sound of the pulses was provided.

### Estimation of learning rates

A reinforcement learning (RL) model was utilized to depict the learning processes in both the active and sham groups. Building upon our prior research (7,12,13), we hypothesized that participants hold a subjective standard in the mind regarding what constitutes a fair split ratio. This standard can be flexibly adjusted based on interaction histories with partners. The internal representation of the fairness norm is updated according to reinforcement learning rules, namely the process of normative adaptation. The hierarchical Bayesian estimator was used, which enabled a simultaneous estimation of individual and group-level learning parameters, one focal free parameter is the learning rate α, which captures how fast one learns and adjusts one's norm. When α is large, individuals can rapidly adjust their fairness norms to adapt to new environments; whereas when α is small, individuals respond more slowly to new information and tend to maintain their original fairness perceptions. See Equation (1).

The computational model was built and estimated using R (version 4.4.0) and the Rstan package (7). The following formula was specified to depict three-stage cognitive process underlying fairness-based decision-making in the UG: the norm adjustment process (Equation (1)), the valuation process (Equation (2)), and the selection process (Equation (3)).

$$fairnorm=fairnorm+ \alpha\times\left( \frac{\gamma\left( responder \right)}{\gamma\left( proposer \right)+\gamma\left( responder \right)}-fairnorm \right) (1)$$

Norm adjustment process: $\gamma\left( responder \right) and \gamma\left( proposer \right)$denote the monetary amounts allocated to the responder and proposer, respectively. $fairnorm$ represents the subjectively expected fair split.

$$Gain\left( accept \right)=\left( 1-\beta\right)\times\gamma\left( responder \right)+ \beta\times\gamma\left( responder \right)\times\left( \frac{\gamma\left( responder \right)}{\gamma\left( proposer \right)+\gamma\left( responder \right)}-fairnorm \right) (2)$$

Valuation Process: The utility of accepting an offer in the Ultimatum Game (UG) is computed as a weighted combination of monetary gain and fairness concern, formalized using a modified Fehr-Schmidt inequality aversion model (Equation (2)). The parameter $\beta$ represents fairness sensitivity, which govers the trade-off between pure monetary utility $\left( 1-\beta\right)\times\gamma\left( responder \right)$ and fairness-related disutility, which scales with the deviation from the expected norm. Greater $\beta$ values imply stronger aversion to inequitable offers, promoting rejection even at personal cost.

$$p\left( acc \right)= \xi\times\frac{exp(\tau\times Gain(acc))}{\exp\left( \tau\times Gain\left( acc \right) \right)+exp(\tau\times Gain(rej))} +\frac{1- \xi}{2} (3)$$

Decision Selection Process: The probability of accepting an offer is modeled through a softmax function with added decision noise: where $Gain(rej)$ = 0 (rejecting yields zero payoff). Here, $\tau$ quantifies decision determinism—higher values increase sensitivity to utility differences, whereas lower values introduce randomness. The term $\xi$ accounts for occasional stochastic errors. This formulation captures bounded rationality and explains inter-individual variability in UG behavior, such as why some subjects reject unfair offers despite monetary loss, while others adaptively accept them.

### Effective connectivity analyses

### *GLM analysis and group-level brain activation detection*

After preprocessing of all the baseline and post-treatment task-based neuroimaging data, a standard GLM analysis implemented in SPM12 was performed to localize brain regions underpinning the neural processing. At the first level analysis, the design matrix was composed of eight regressors, including two regressors of the fair offer and unfair offer regressors and six regressors of head motion related parameters in the realignment of fMRI sequence. Then, contrasts of interest (i.e., [unfair+fair] offer condition; [unfair-fair] offer condition) were entered into a second-level random-effect analysis to detect group-level effects. We firstly conducted a one-sample t-test for the [unfair+fair] contrasts to detect the regions showing significant activities during the offer presentation across all the participants (voxel-level *p* = 0.001, cluster-level FWE *p* < 0.05). Then, using these regions as a mask, we conducted a 2×2 flexible design analysis for the [unfair-fair] contrasts to detect the regions whose activity was modulated by group, or time, or their interaction. The brain regions showing significant interaction effects constitute our primary focus (voxel-level *p* = 0.005, > 30 voxels).

These regions identified through the above interaction effects were used as volumes of interest (VOI) in the subsequent dynamic causal modeling (DCM) analysis. Meanwhile, considering the insular’s important role in the decision process of the UG task, the bilaterial insula identified during the offer presentation stage were also included. The group-level VOI coordinates are shown in Table S1. To extract time series of VOIs, we searched for the local maximum within the group-level VOI and summarized the regional response with the first eigenvariate of (confound-corrected) voxels within an 8 mm radius.

***Dynamic causal modeling***

We specified a determined DCM for each participant. In this model, all the connections within and between regions were specified, which are the average connectivity during the offer presentation stage. The modulatory effects of experimental manipulation on all the connections were from the inputs of the offer fairness level. The main effect of task (the offer events) entered the model as driving inputs. Then, each participant’s model was inverted, providing estimates of the connection strength parameters.

We used Parametric Empirical Bayes (PEB) to estimate the group mean and the effects of group, time and group×time interaction for each connection (14). This routine has been used in one of our previous studies (15). In brief, PEB is a between-subjects hierarchical or empirical Bayesian model over parameters that models how individual (within-subject) connections relate to group or condition means. This hierarchical model treats each connection as a random (between-subjects) effect, which is modeled by adding a random Gaussian component to subject-specific parameters, based upon the group mean connectivity as well as between-subject effects. This random effect modeling uses the full posterior density over the parameters from each subject**’**s DCM**—**both the expected strength of each connection and the associated uncertainty (i.e., posterior covariance)**—**to inform the group-level result.

This study aims to find the cTBS treatment effect on effective connectivity and test whether the changes in effective connectivity in the cTBS group is different from that in the sham group. Therefore, we set four covariates of interest in the design matrix of Bayesian GLM (PEB model) for average connectivity (matrix A) and task modulation parameters (matrix B), separately: the group mean (the default), group, time and the interaction between group and time. The connections showing significant interaction effects constitute our primary focus.

To evaluate the above-mentioned effects on connections, we used Bayesian Model Reduction to search over hundreds of reduced PEB models with different combinations of connections. The reduced PEB models were obtained by removing one or more (second level) parameters from the full PEB model (described above) to produce reduced forms of the full model that differed only in their priors. By iteratively pruning connection parameters from the full PEB model, the Bayesian Model Reduction provides an efficient search of the model space by scoring each reduced model, based on its log model-evidence or free energy; for details, see (14). The best 256 pruned models from this search were combined using Bayesian Model Averaging. These averages were reported in the results. We reported the results in a threshold of free energy with or without and posterior probability (*Pp*) > 0.95.

1. **Supplementary Results**

**Effective connectivity results**

Figure S2 shows group differences in connectivity. The patients in the cTBS group had decreased inhibitory self-connection in the ACC and decreased excitatory connection of the L.Insula→ACC compared with the sham group.

Figure S3 shows time changes in connectivity. At the endpoint of 2-week treatment, the inhibitory self-connection in the L.DLPFC, the excitatory connections of the ACC→R.Insula and the L.MOG→ACC were decreased, while the excitaItory connections of the R.Insula→bilateral DLPFC were increased.

Figure S2. The connections modulated by group effects and the effective connectivity strengths plotted in each group and time point. L, left; R, right; EC, effective connectivity.

Figure S3. The connections modulated by time effects and the effective connectivity strengths plotted in each group and time point. L, left; R, right; EC, effective connectivity.

1. **Supplementary Discussion**

### Improvement in social cognition

We observed that the changes in learning rates did not align with changes in the acceptance rates in both groups, suggesting the markedly difference between cognitive learning abilities and social behaviors. Learning rates are more directly related to cognitive functions, specifically the leaning abilities of adapting to new information based on prior knowledge, while the acceptance of unfair offers may be more influenced by emotional, social interaction, and response inhibition in the decision. Notably, hierarchical Bayesian behavioral modeling provides enhanced sensitivity in quantifying latent cognitive processes compared to conventional simple behavioral analyses (e.g., acceptance rates), enabling more precise detection of subtle yet reliable cognitive changes (16,17). These findings collectively suggest that integrating measurements across both social cognitive processing and social decision-making behaviors provides more comprehensive evidence for evaluating the impact of individualized right DLPFC-targeted TMS on social cognition and social function.

### Placebo effects in the sham group

Notably, our sham control group also exhibited significant improvements in both clinical symptoms and general social functioning. This observation aligns with established evidence demonstrating robust placebo effects in rTMS trials for major depressive disorder (18–20). A comprehensive meta-analysis of 61 sham-controlled TMS studies quantified this effect, revealing a substantial placebo response (Hedges’g = 0.8) when comparing baseline to post-treatment depression scores in sham groups (20). Several factors may contribute to this phenomenon: (1) the non-specific therapeutic effects of increased clinical attention and patient expectations (21), and (2) potential natural symptom fluctuation during the study period. These findings carry important clinical implications. The demonstrated placebo response, coupled with the active treatment effects, suggests that combining rTMS with structured psychological interventions may yield superior therapeutic outcomes. Future trials should consider incorporating integrated treatment protocols to capitalize on both neuromodulation and psychosocial therapeutic mechanisms.

### Table S1. The list of the volumes of interest and group-level coordinates

| VOI | MNI coordinates | | |
| --- | --- | --- | --- |
|  | x | y | z |
| ACC | -3 | 18 | 57 |
| L.DLPFC | -45 | 24 | 39 |
| R.DLPFC | 54 | 30 | 33 |
| L.Insula | -33 | 18 | -6 |
| R.Insula | 33 | 21 | -3 |
| L.MOG | -33 | -90 | -3 |
| R.MOG | 33 | -87 | -6 |

### Table S2. Adverse effects

| Adverse effects | Active group  (n = 33) | sham group  (n = 30) | χ^2^ | *p* |
| --- | --- | --- | --- | --- |
| Localized pain | 4 | - | 3.88 | 0.049^*^ |
| Teeth trembling | 3 | - | 2.86 | 0.091 |
| Headache | 1 | 1 | 0.01 | 0.945 |
| Dizziness | - | 2 | 2.27 | 0.132 |
| Change in sleepiness | - | 2 | 2.27 | 0.132 |
| Facial muscle twitching | 1 | - | 0.92 | 0.336 |

^*^*p* < 0.05

### Table S3. Correlations between effective connectivity changes and clinical/behavioral improvement

| Clinical/behavioral Measures | R.Insula→R.DLPFC | | ACC→L.Insula | |
| --- | --- | --- | --- | --- |
|  | *r* | *p* | *r* | *p* |
| **Active group** | | | | |
| HAMD-17 | 0.025 | 0.898 | -0.195 | 0.310 |
| HAMA | 0.036 | 0.852 | -0.063 | 0.747 |
| GAF | 0.101 | 0.603 | 0.207 | 0.280 |
| Acceptance rate | 0.210 | 0.275 | -0.234 | 0.222 |
| Learning rate | 0.303 | 0.111 | -0.317 | 0.094 |
| **Sham group** | | | | |
| HAMD-17 | 0.292 | 0.176 | -0.257 | 0.237 |
| HAMA | 0.151 | 0.492 | -0.179 | 0.439 |
| GAF | 0.210 | 0.336 | -0.304 | 0.158 |
| Acceptance rate | -0.015 | 0.945 | -0.082 | 0.709 |
| Learning rate | -0.259 | 0.232 | 0.305 | 0.157 |

1. **Supplementary References**

1. Martin DM, McClintock SM, Forster JJ, Lo TY, Loo CK (2017): Cognitive enhancing effects of rTMS administered to the prefrontal cortex in patients with depression: A systematic review and meta-analysis of individual task effects. *Depress Anxiety* 34: 1029–1039.

2. Iimori T, Nakajima S, Miyazaki T, Tarumi R, Ogyu K, Wada M, *et al.* (2019): Effectiveness of the prefrontal repetitive transcranial magnetic stimulation on cognitive profiles in depression, schizophrenia, and Alzheimer’s disease: A systematic review. *Prog Neuropsychopharmacol Biol Psychiatry* 88: 31–40.

3. Gabay AS, Radua J, Kempton MJ, Mehta MA (2014): The Ultimatum Game and the brain: a meta-analysis of neuroimaging studies. *Neurosci Biobehav Rev* 47: 549–558.

4. Feng C, Luo Y-J, Krueger F (2015): Neural signatures of fairness-related normative decision making in the ultimatum game: a coordinate-based meta-analysis. *Hum Brain Mapp* 36: 591–602.

5. Jin Y, Gao Q, Wang Y, Xiao L, Wu MS, Zhou Y (2022): The perception-behavior dissociation in the ultimatum game in unmedicated patients with major depressive disorders. *J Psychopathol Clin Sci* 131: 253–264.

6. Wang Y, Zhou Y, Li S, Wang P, Wu G-W, Liu Z-N (2014): Impaired social decision making in patients with major depressive disorder. *BMC Psychiatry* 14: 18.

7. Jin Y, Zheng D, Gu R, Fan Q, Dietz M, Wang C, *et al.* (2025): Substantial Heritability Underlies Fairness Norm Adaptation Capability and its Neural Basis. *Adv Sci Weinh Baden-Wurtt Ger* 12: e2411070.

8. Hasler G (2012): Can the neuroeconomics revolution revolutionize psychiatry? *Neurosci Biobehav Rev* 36: 64–78.

9. Güth W, Schmittberger R, Schwarze B (1982): An experimental analysis of ultimatum bargaining. *J Econ Behav Organ* 3: 367–388.

10. Gradin VB, Pérez A, MacFarlane JA, Cavin I, Waiter G, Engelmann J, *et al.* (2015): Abnormal brain responses to social fairness in depression: an fMRI study using the Ultimatum Game. *Psychol Med* 45: 1241–1251.

11. Zhao N, Yue J, Feng Z-J, Qiao Y, Ge Q, Yuan L-X, *et al.* (2022): The Location Reliability of the Resting-State fMRI FC of Emotional Regions Towards rTMS Therapy. *Neuroinformatics* 20: 1055–1064.

12. Gu X, Wang X, Hula A, Wang S, Xu S, Lohrenz TM, *et al.* (2015): Necessary, yet dissociable contributions of the insular and ventromedial prefrontal cortices to norm adaptation: computational and lesion evidence in humans. *J Neurosci Off J Soc Neurosci* 35: 467–473.

13. Hétu S, Luo Y, D’Ardenne K, Lohrenz T, Montague PR (2017): Human substantia nigra and ventral tegmental area involvement in computing social error signals during the ultimatum game. *Soc Cogn Affect Neurosci* 12: 1972–1982.

14. Friston KJ, Litvak V, Oswal A, Razi A, Stephan KE, van Wijk BCM, *et al.* (2016): Bayesian model reduction and empirical Bayes for group (DCM) studies. *NeuroImage* 128: 413–431.

15. Zhou Y, Zeidman P, Wu S, Razi A, Chen C, Yang L, *et al.* (2018): Altered intrinsic and extrinsic connectivity in schizophrenia. *NeuroImage Clin* 17: 704–716.

16. Gershman SJ (2016): Empirical priors for reinforcement learning models. *J Math Psychol* 71: 1–6.

17. Lieder F, Griffiths TL (2019): Resource-rational analysis: Understanding human cognition as the optimal use of limited computational resources. *Behav Brain Sci* 43: e1.

18. Burke MJ, Romanella SM, Mencarelli L, Greben R, Fox MD, Kaptchuk TJ, *et al.* (2022): Placebo effects and neuromodulation for depression: a meta-analysis and evaluation of shared mechanisms. *Mol Psychiatry* 27: 1658–1666.

19. Burke MJ, Kaptchuk TJ, Pascual-Leone A (2019): Challenges of differential placebo effects in contemporary medicine: The example of brain stimulation. *Ann Neurol* 85: 12–20.

20. Razza LB, Moffa AH, Moreno ML, Carvalho AF, Padberg F, Fregni F, Brunoni AR (2018): A systematic review and meta-analysis on placebo response to repetitive transcranial magnetic stimulation for depression trials. *Prog Neuropsychopharmacol Biol Psychiatry* 81: 105–113.

21. Morberg BM, Malling AS, Jensen BR, Gredal O, Wermuth L, Bech P (2018): The Hawthorne effect as a pre-placebo expectation in Parkinsons disease patients participating in a randomized placebo-controlled clinical study. *Nord J Psychiatry* 72: 442–446.
